# Transcriptomic Analysis of the Developing Testis and Spermatogenesis in Qianbei Ma Goats

**DOI:** 10.3390/genes14071334

**Published:** 2023-06-25

**Authors:** Yue Zou, Xiang Chen, Xingzhou Tian, Wei Guo, Yong Ruan, Wen Tang, Kaibin Fu, Taotao Ji

**Affiliations:** 1 Key Laboratory of Animal Genetics, Breeding and Reproduction in the Plateau Mountainous Region, Ministry of Education, Guizhou University, Guiyang 550025, China; zoekiwi@163.com (Y.Z.); tianxingzhou@yeah.net (X.T.); guow16@lzu.edu.cn (W.G.); yruan@gzu.edu.cn (Y.R.); went4863@gmail.com (W.T.); kbinfu@163.com (K.F.); taotaoji20@163.com (T.J.); 2 Key Laboratory of Animal Genetics, Breeding and Reproduction, Guiyang 550025, China; 3 College of Animal Science, Guizhou University, Guiyang 550025, China

**Keywords:** Qianbei Ma goat, testis development, RNA-Seq, mRNA expression

## Abstract

Reproductive competence in male mammals depends on testicular function. Testicular development and spermatogenesis in goats involve highly complex physiological processes. In this study, six testes were, respectively, obtained from each age group, immature (1 month), sexually mature (6 months) and physically mature (12 months old) Qianbei Ma goats. RNA-Seq was performed to assess testicular mRNA expression in Qianbei Ma goats at different developmental stages. Totally, 18 libraries were constructed to screen genes and pathways involved in testis development and spermatogenesis. Totally, 9724 upregulated and 4153 downregulated DEGs were found between immature (I) and sexually mature (S) samples; 7 upregulated and 3 downregulated DEGs were found between sexually mature (S) and physically mature (P) samples, and about 4% of the DEGs underwent alternative splicing events between I and S. Select genes were assessed by qRT-PCR, corroborating RNA-Seq findings. The detected genes have key roles in multiple developmental stages of goat testicular development and spermatogenesis. Gene Ontology (GO) and Kyoto Encyclopedia of Genes and Genomes (KEGG) analyses were performed to determine differentially expressed genes (DEGs). GO analysis revealed DEGs between S and P contributed to “reproduction process”, “channel activity” and “cell periphery part” between I and S, and in “ion transport process”, “channel activity” and “transporter complex part”. KEGG analysis suggested the involvement of “glycerolipid metabolism”, “steroid hormone biosynthesis” and “MAPK signaling pathway” in testis development and spermatogenesis. Genes including *IGF1*, *TGFB1*, *TGFBR1* and *EGFR* may control the development of the testis from immature to sexually mature, which might be important candidate genes for the development of goat testis. The current study provides novel insights into goat testicular development and spermatogenesis.

## 1. Introduction

The Qianbei Ma goat is a unique goat breed in the mountainous area of Guizhou Plateau. It is large in size, strong in constitution and delicious in taste. It is a goat breed whose skin and meat are both valuable, and has many advantages such as strong adaptability, high fertility, stable genetic inheritance and strong disease resistance. However, the research on its reproductive performance mainly focuses on lambing and estrus in she-goat and the molecular biology understanding of testicular development or spermatogenesis in Qianbei Ma goat is very limited, deserving further investigation. The breeding performance of male animals is also important to the total fertility of the population. Additionally, the most important guarantee for the reproductive performance of rams is the normal development of testis and spermatogenesis. Testicular development and spermatogenesis are very complex and precise processes which are regulated by multiple genes.

The testis represents a critical organ of the male reproductive system in mammals, producing spermatozoids and androgens. Spermatogenesis constitutes a developmental event, which produces haploid spermatozoa from diploid spermatogonial stem cells during meiosis in the testis [1]. Spermatogenesis comprises three steps, including spermatogonial proliferation and differentiation, meiotic division of spermatocytes and spermatozoid maturation [2]. Aside from spermatogenic cells, spermatogenic mechanisms involve many somatic cells in the testicle, including Sertoli and Leydig cells. In the testis of mammals, spermatogenesis occurs in seminiferous tubules, where germ cells are associated with Sertoli cells. Genes associated with the latter cells also have critical functions in precise steps of spermatogenesis [3]. The proper development of the testicles ensures the spermatogenesis process.

RNA sequencing (RNA-Seq) allows an expression profiling of genes and might help map and quantify the transcriptome [4,5]. This approach offers many advantages compared to other transcriptomic tools, including high resolution/sensitivity, a broad dynamic range of gene expression, and the identification of new transcript sequences and splice isoforms of previously reported genes [6]. Ramsköld [7] evaluated multiple tissues from mammals by RNA-Seq and reported that most genes were specifically expressed in testicular samples. Additionally, considering RNA-Seq-based expression patterns, Djureinovic [8] categorized 20,050 putative human genes, which showed specific expression in the human testicle. Their evaluation revealed the testicular tissue had by far the largest quantity of tissue-specific genes. Using microarray analysis, Anand detected differentially expressed genes (DEGs) in testicular samples compared to other tissues, identifying 2868 upregulated transcripts and 2011 downregulated mRNAs [9]. The testicle appears to have a higher degree of metabolic activity relative to other normal tissues. Most current reports assessing the association of testicular development with spermatogenesis have been performed in the human or mouse species, and the goat is scarcely examined.

The present work aimed to perform transcriptome profiling of immature and mature Qianbei Ma goat testis specimens by RNA-Seq and bioinformatic analysis. The findings provide novel insights into the mechanisms regulating goat testicular development and spermatogenesis.

## 2. Materials and Methods

### 2.1. Ethics Statement

This study had approval from the Animal Ethics Committee of Guizhou University (Guiyang, China) (No. EAE-GZU-2021-P024, Guiyang, China; 30 March 2021).

### 2.2. Animal Handling and Sample Collection

Permission was granted to castrate eighteen healthy Qianbei Ma goats in Fuxing Husbandry Co., Ltd., Zunyi, Guizhou, China. Goat ages were obtained from goat farming records. There were six immature goats (1 month old, before sexual maturation, i.e., samples I1, I2, I3, I4, I5 and I6), six sexually mature goats (6 months old, after sexual maturation but before physical maturation, i.e., samples S1, S2, S3, S4, S5 and S6) and six physically mature goats (12 months old, after physical maturation, i.e., samples P1, P2, P3, P4, P5 and P6). We surgically collected the right testes from the eighteen goats by castration after anesthesia, followed by storage in RNA/DNA sample protector (Servicebio, Wuhan, China). The testis from each goat was cut longitudinally, and a small amount (3–5 g) of the parenchyma, including seminiferous tubules and Leydig cells, underwent snap freezing in liquid nitrogen and was transported to the lab for further studies. All castrated goats remained in Fuxing Husbandry Co., Ltd. (Guizhou, China) after our study, for fatten feeding.

### 2.3. RNA Quantitation and Quality 

Total RNA extraction was carried out from the testicular tissue in groups I, S and P using TRIzol reagent (Servicebio, Wuhan, China) and RNeasy RNA purification kit (Servicebio, Wuhan, China) with DNase as directed by the manufacturer. A NanoDrop™ One spectrophotometer (Thermo Fisher Scientific, Waltham, Ma, USA) was utilized to assess RNA purity and amounts. RNA quality assessment utilized 1% agarose gel electrophoresis. High-quality RNA samples (OD 260/280 of 1.8–2.0, integrity > 7.0 and 28S:18S above 1.0) were sequenced on an Illumina NovaSeq 6000 system, generating 150-bp paired end reads.

### 2.4. Transcriptome Sequencing

Approximately 5 μg RNA/sample constituted the input material for RNA sample preparation. Index-coded samples were clustered with NEBNext^®^ Ultra™ Directional RNA Library Prep Kit for Illumina^®^ according to a protocol provided by the manufacturer. Upon clustering, the prepared libraries underwent sequencing on an Illumina NovaSeq 6000 (Illumina, San Francisco, CA, USA). The image data of the sequences yielded by the high-throughput sequencer were converted into sequence data (reads) by CASA V A base recognition to obtain FASTQ files. Raw RNA-Seq FASTQ data next underwent filtration with Fastp v [10], a quality control software that can quickly filter and correct FASTQ data to exclude adapter-containing, N-containing and low-quality (quality score below 20) reads, resulting in clean reads, and were mapped to the goat (*C. hircus*) (ARS1.2) reference genome [4] using HISAT2, a software uses a graph Ferragina Manzini index to align DNA and RNA sequences [11].

### 2.5. Quantification of Gene Expression

Reads mapped to a given gene were counted with featurerts for estimating the expression of various gene transcripts. Gene expression was determined from million mapped reads per kilobase (FPKM) values [12], the current commonest approach to estimate gene expression [13].

### 2.6. Differential Expression Analysis

The DESeq2 software (1.20.0) was used to analyze differential expression between the treatment and control groups. The Benjamini–Hochberg algorithm was utilized to adjust *p* values (p-adj) to control for false discovery rate. |log2 (FoldChange)| ≥ 1 and padj < 0.05 was set as the significance threshold for differential expression [14].

### 2.7. GO and KEGG Enrichment Analyses of DEGs

GO and KEGG analyses of DEGs were implemented with ClusterProfiler, correcting for gene length bias. KEGG is an information database based on molecular findings, particularly via genome sequencing and additional high-throughput techniques to produce large-scale molecular data sets, allowing a deep understanding of biological systems (http://www.genome.jp/kegg/, accessed on 21 July 2022) [15]. GO and KEGG terms with |log2 (FoldChange)| ≥1 and padj < 0.05 were deemed to be DEGs with significant enrichment [10].

### 2.8. Prediction of New Transcripts and Alternative Splicing Analysis

StringTie was utilized to build and identify previously reported and new transcripts from HISAT2 alignment data. StringTie utilizes a network-flow algorithm with optional de novo assembly to splice transcripts. Compared with cufflinks, StringTie has the following classifying AS events, which were assessed in various samples separately.

Advantages: (1) it yields more complete transcripts; (2) assembles more accurate transcripts; (3) better estimates the transcript’s expression level and (4) has greater splicing speed [16,17]. rMATS (http://rnaseqmats.sourceforge.net/index.html, accessed on 22 July 2022) was utilized to classify events.

### 2.9. qRT-PCR for RNA-Seq Data Validation

By analyzing the results of RNA sequencing and combining with the results of other earlier studies [18,19,20,21,22,23], we screened *TGFBR1*, *TGFB1*, *EGFR*, *IGF1*, *MAPK3* and *SMAD4* associated with testicular development or spermatogenesis. These DEGs were significantly up-regulated or down-regulated in our RNA sequencing results. The six selected DEGs were examined by qRT-PCR to validate the correctness of our RNA-Seq findings. Total RNA (1000 ng) was utilized to produce complementary DNA (cDNA) with 2×SYBR Green qPCR Master Mix None ROX (Servicebio, Wuhan, China) at 25 °C (5 min), 42 °C (30 min) and 85 °C (5 s). The primers used for qRT-PCR are shown in Table 1. A CFX96 Real-Time PCR system (Bio-Rad, Hercules, CA, USA) was utilized for amplification in 15-µL reactions containing 2×qPCR Mix (7.5 µL), forward and reverse primers (10 pmol/µL, 0.75 µL each), cDNA (1000 ng/µL, 2 µL) and nuclease-free water (4 µL). The reaction conditions were: 1 cycle at 95 °C (30 s), followed by 40 cycles at 95 °C (15 s) and 60 °C (30 s), with fluorescence signals collected every 0.5 °C increase from 65 °C to 95 °C. Melting curves were utilized to assess primer specificity. Assays were carried out in triplicate and *GAPDH* was utilized for normalization in data analysis by the 2^−∆∆Ct^ method.

## 3. Results

### 3.1. Gene Expression Profiling during Testicular Development in Qianbei Ma Goats

To determine the genes associated with testicular development and sperm formation, 18 libraries, including 6 each from immature, sexually mature and physically mature testes were sequenced. Table 2 shows an overview of the information pertaining to raw and clean reads for all libraries. Error rates and GC contents for various libraries were determined for their quality control. All 18 libraries were of high quality.

Paired-end clean reads underwent alignment to the goat reference genome ARS1.2 that is commonly utilized for *C. hircus* (goats) [4] with HISAT2, and the expected number of fragments per kilobase of transcript sequence per million base pairs sequenced (FPKM) of each gene was calculated. Table 3 shows an overview of the information about uniquely mapped clean reads.

Additionally, an overview of the percentages of clean reads mapped to exon borders (junction reads) is shown in Table 4.

All RNA-Seq data in our study had been submitted to NCBI (accession number BioProject: PRJNA879963).

### 3.2. Alternative Splicing Data

In this work, AS events were categorized into five types with rMATS (http://rnaseq-mats.sourceforge.net/index.html, accessed on 21 July 2022). By determining the types and amounts of AS events, and analyzing each AS type, a large number of AS events were found in testicular development and sperm formation. Of all the DEGs detected between immature (I) and sexually mature (S) testes in our present work, 544 genes underwent AS events between sexually mature (S) and physically mature (P) testes, with no detected AS events (padj ≤ 0.05). Therefore, approximately 4% of genes showed AS between I and S, and no gene had AS between S and P in this study. The above findings indicated AS was very important in the complexity of gene expression during testis development, especially in the period from immaturity to sexual maturity.

**Figure 1:**
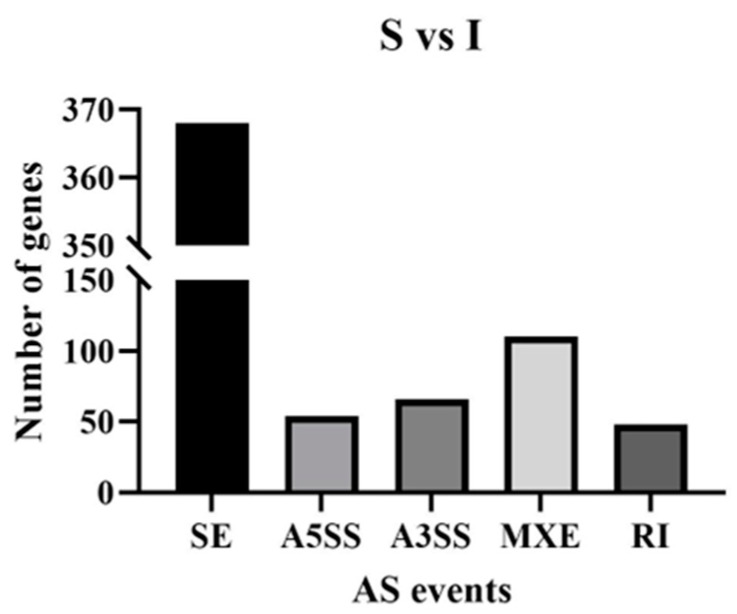
AS events among genes for immature and sexually mature group comparisons. X axis represents five known types of AS events, respectively, skipped exon (SE), alternative 5′ splice site (A5SS), alternative 3′ splice site (A3SS), mutually exclusive exon (MXE) and retained intron (RI).

To determine AS types associated with testicular genes, AS events were compared between the S group and the I group. The five known types of AS events include retained intron (RI), mutually exclusive exon (MXE), alternative 3′ splice site (A3SS), alternative 5′ splice site (A5SS) and skipped exon (SE). All five types of AS were found in the S vs. I group comparison. RI, MXE, A3SS and SE were found in the P vs. S group comparison. These findings showed SE as the most common AS event amounting to 368 in S vs. I. Other identified AS events were RI (48), MXE (110), A3SS (66) and A5SS (54) (Figure 1).

### 3.3. Analysis of DEGs

**Figure 2:**
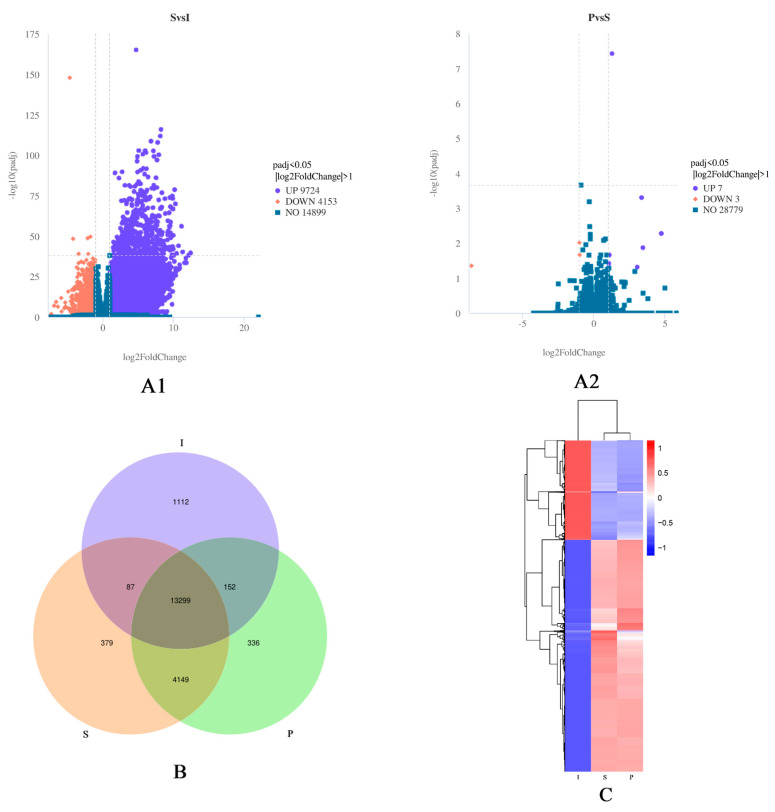
(**A1**): Volcanic plot of differentially expressed genes (DEGs) between the immature and sexually mature groups. (**A2**): Volcanic plot of DEGs between the sexually mature and physically mature groups. Highly significant differences in the expression of up− (purple) and down− (red) regulated genes were observed between immature (I) and sexually mature (S) testes, and between sexually mature (S) and physically mature (P) testes. Blue indicates no differential expression. (**B**): Venn diagram depicting gene expression patterns. The numbers of uniquely and commonly (FPKM > 1) expressed genes were shown. (**C**): Clustering of differentially expressed genes. The overall FPKM hierarchical clustering map was obtained with log10 (FPKM + 1) for normalization. Different columns represent groups, different rows represent genes, different colors represent expression levels (red: high, blue: low).

In order to clarify the difference in gene expression in testis of Qianbei Ma goat at different developmental stages, we compared and analyzed the differentially expressed genes in three different developmental stages. DEGs were determined with |log2 (fold−change)| ≥ 1 and padj < 0.05. As a result, a total of 9,724 upregulated and 4,153 downregulated DEGs were detected between the I and S groups; 7 upregulated and 3 downregulated genes were detected between the S and P groups (Figure 2A1, Figure 2A2). Among all DEGs detected, 1112 genes were only expressed in the immature group, 379 genes were only expressed in the sexual mature group, 336 genes were only expressed in the physical mature group, and 13,299 genes were expressed in all three groups (Figure 2B). Figure 2C depicts hierarchical clustering, with DEGs for the eighteen libraries grouped into three clusters, the result suggested the I, S and P groups had differential expression patterns overall, but S and P had similar repetitive expression commonalities.

### 3.4. GO Analysis of DEGs

**Figure 3:**
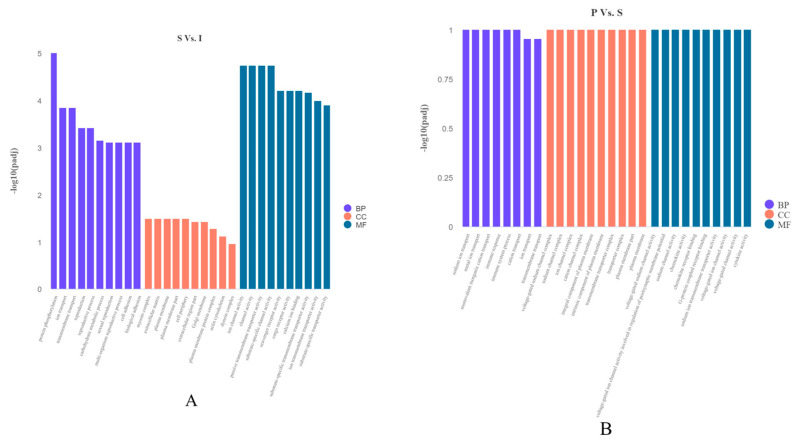
(**A**): Most enriched GO terms between immature (I) and sexually mature (S) testes. (**B**): Most enriched GO terms between sexually mature (S) and physically mature (P) testes. Abscissas and ordinates represent enriched GO terms and significance levels of GO enrichment, respectively. Purple, “biological processes”; red, “cellular components”; blue, “molecular functions”.

GO analysis was carried out to examine the functions of DEGs in testicular development. Totally, 106 GO terms associated with “biological processes”, “molecular functions” and “cellular components” were markedly enriched between immature (I) and sexually mature (S) testes. We found 43 GO terms belonged to “biological process”, 56 GO terms belonged to “molecular functions”, and 7 GO terms belonged to “cellular components”. Totally, 36 GO terms associated with “biological processes”, “molecular functions” and “cellular components” had significant enrichment in the sexually mature (S) vs. physically mature (P) testes. We found 5 GO terms belonged to “biological processes”, 21 GO terms belonged to “molecular functions”, and 10 GO terms belonged to “cellular components” (Table 5, Figure 3A,B).

### 3.5. KEGG Pathway Analysis of DEGs

**Figure 4:**
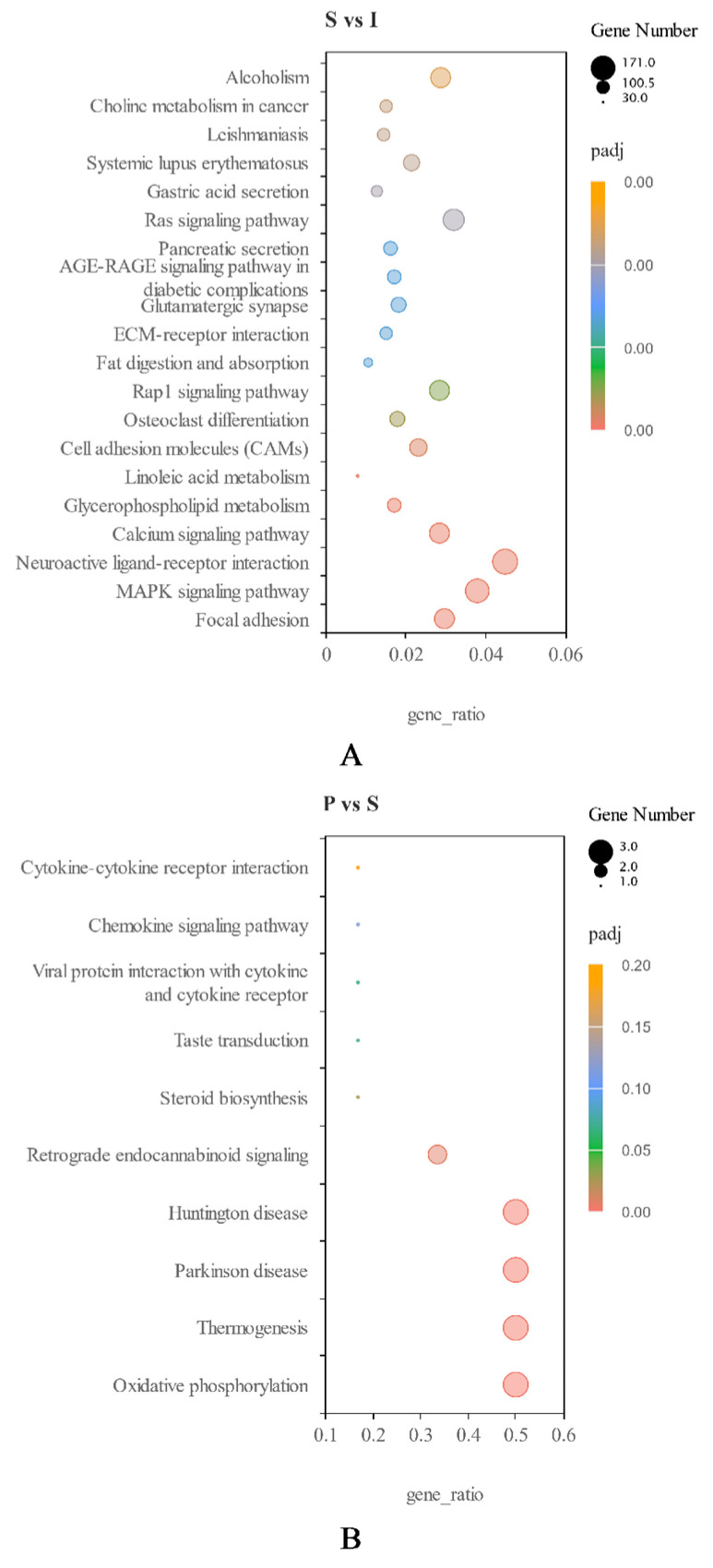
(**A**): Scatter plot of differentially expressed KEGG genes in immature (I) and sexually mature (S) testes. (**B**): Scatter plot of differentially expressed KEGG genes in sexually mature (S) and physically mature (P) testes. Ordinates and abscissas represent the names of KEGG pathways and gene ratios, respectively. Point sizes and colors represent the numbers of DEGs and the ranges of Q values, respectively.

KEGG pathway analysis of DEGs (|log2 (fold−change)| ≥ 1 and padj < 0.05) was carried out. For I vs. S, 8 KEGG pathways were upregulated, including “glycerolipid metabolism”, “protein digestion and absorption” and “steroid hormone biosynthesis”. Totally, 90 pathways were downregulated, including +“lysosome”, “MAPK signaling pathway” and “chemokine signaling pathway” (Figure 4A). For S vs. P, 7 KEGG pathways were upregulated, including “oxidative phosphorylation”, “thermogenesis” and “steroid biosynthesis”. However, no pathways were significantly downregulated (Figure 4B).

### 3.6. qRT-PCR Validation of DEGs

**Figure 5:**
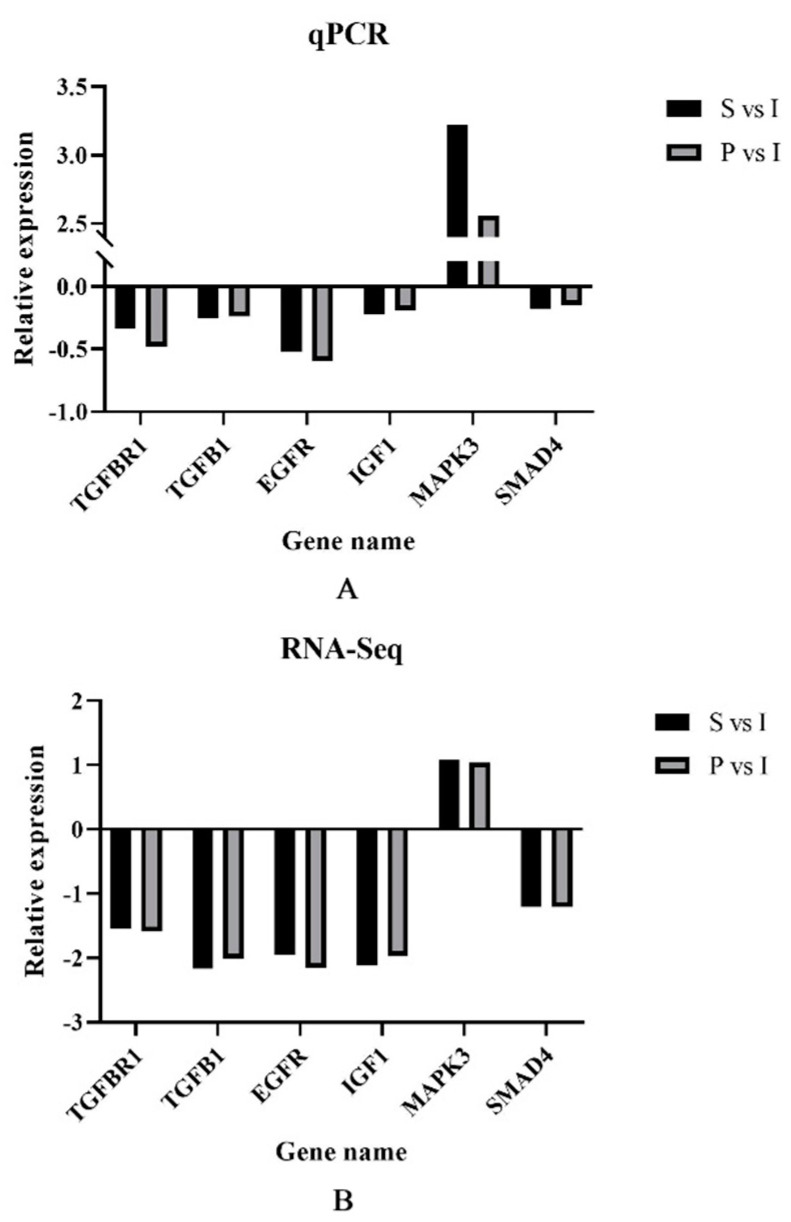
Relative expression of different genes in sexually mature (S) and physically mature (P) testes compared to immature (I) testes, respectively, using group I as a reference. (**A**): qPCR relative expression of S vs. I and P vs. I. (**B**): RNA−Seq data for S vs. I and P vs. I.

To verify the DEGs in immature (I), sexually mature (S) and physically mature (P) testes, we selected 6 DEGs, including *TGFBR1*, *TGFB1*, *EGFR*, *IGF1*, *MAPK3* and *SMAD4*, to validate RNA-Seq data by qRT-PCR. qRT-PCR data corroborated RNA-Seq findings, suggesting the reliability of RNA-Seq data (Figure 5).

## 4. Discussion

With the current development of detection technology, more and more mRNAs on testis related to testis development and spermatogenesis have been reported. RNA-Seq has emerged as a tool for efficiently and inexpensively detecting new transcripts and genes. RNA-Seq methods have been broadly utilized to determine DEGs or gene expression patterns, new transcripts, AS events and SNPS, and have empowered studies examining porcine [24,25], cattle [26,27] and mouse [28,29] testicular development. In goats, the profiles of ovarian [30,31], uterine [32,33] and testicular [34,35] tissues under different conditions were recently compared by RNA-Seq. However, limited data on testicular development in goats were available. Breed and age represent major factors affecting testicular development. Here, RNA-Seq was performed to build a complete dataset that explains the spatiotemporal transcriptome of the testicular tissue in Qianbei Ma goats. Testicular growth and development constitute the key factors affecting goat reproduction. Therefore, identifying genes regulating testicular growth and development is critical. In this study, 13,887 genes were assessed by RNA-Seq in six immature, six sexually mature and six physically mature testes. Totally, 9724 genes were upregulated and 4153 were downregulated between immature and sexually mature testes; seven genes were upregulated and three were downregulated between sexually mature and physically mature testes which was similar to the hierarchical clustering analysis result in our study. The reason for this result is related to the development process of the testis of Qianbei Ma goat. We speculate that the testis develops from immature to sexual mature with various reproductive functions and also starts to produce sperm, which is a very complex process that requires the co-regulation of a large number of different genes. When the goats continue to develop from sexual maturity to physical maturity, the testes already had all the functions related to reproduction, so the differences in development were relatively small, the number of related DEGs was significantly reduced, which was similar to the results of Gong studies on testis of mice at three different developmental stages [36]. Using next-generation platforms, we determined most upregulated genes were associated with protein coding and might have functions in testicular development and sperm formation.

Alternative splicing (AS) represents a commonly encountered phenomenon in eukaryotes, which could lead to the production of various protein forms at different times under different circumstances, increasing species/body fitness. Although AS research is a known subfield of molecular biology, only in recent years has this subfield attracted sufficient attention. AS is critical for the complex proteomes and functions found in higher organisms. AS is an important mechanism in the regulation of gene expression and promotes proteome diversity [37]. It is estimated that about 95% of human multiple-exon gene expression is associated with AS events [38]. In metazoans, AS is critical for the production of various protein forms with functions in different cell events such as cell growth, differentiation and death [39]. Here, five AS events were observed, mostly involving ES. The effects of AS events on the functions of related genes can be predicted by a comprehensive analysis of AS events, and GO and KEGG analysis data [40,41]. In the current study, Sec insertion sequence binding protein 2 (*SECISBP2*) was the gene with the highest number of SE events, i.e., a total of ten SE events. *SECISBP2* underwent the most AS events during the development, indicating that SECISBP2 protein synthesis is complex and important to Qianbei Ma goats’ testis development. Mutation of the gene altered thyroid hormone metabolism [42,43]. Thyroid hormones can modulate semen quality under physiological conditions by regulating testosterone and changing some semen indexes [44,45,46]. Thyroid hormone levels in the early stages of testicular development can also influence testicular development and spermatogenesis by regulating the length of time during which supporting cells proliferate [47]. Based on previous studies, we hypothesized that there is active synthesis of SECISBP2 during testicular development in Qianbei Ma goat, which is essential for testicular size and spermatogenesis.

Combining previous relevant reports, and KEGG and GO data in the current study, the genes involved in the regulation of testis development and sperm formation through protein phosphorylation were mainly *TGFB1*, *EGFR* and *IGF1*, which have critical functions in testis growth, hormone secretion, spermatogenesis and Leydig cell differentiation. 

Transforming growth factor β-1 (*TGFB1*) plays multiple biological roles, including the control of proliferative and differentiation potentials of cells in rams [48]. In male lambs, *TGFB1* regulates tight junctions in Sertoli cells and controls spermatogenesis. It modifies the blood-testicular barrier (BTB) by downregulating tight junction proteins [49]. *TGFB1* might play an important role in testicular development because of its high expression in the immature testis and markedly reduced expression in sexual maturity, as spermatogenesis begins. A comparable expression pattern was found for TGFB receptor type 1 (*TGFBR1*) [50]. In addition, loss of *TGFB1* resulted in lower testosterone levels in the testis and serum, and decreased the ability to mate with females [51]. Based on previous studies, we speculated that *TGFB1* may not only directly regulate goat testicular development and sperm formation, but also ensure the normal development of male external genitalia and affect fertility.

Epidermal growth factor receptor (*EGFR*) represents a receptor gene for EGF and controls testicular function in the human, mouse, rat and livestock species as well as in alpacas [20]. In yak and bovine, EGF and *EGFR* were critical paracrine and/or autocrine modulators of testicular development and sperm formation and regulate testosterone production by testicular interstitial cells [52,53]. We speculate that EGF and *EGFR* may also be expressed in various goat testicular cells, stimulate testosterone secretion, and regulate testis development and spermatogenesis.

Insulin-like growth factor I (*IGF1*) contributes to the regulation of testicular function [54]. Pitetti et al. indicated growth factors of the insulin family play essential roles by controlling SC number, testis size and daily sperm production [55,56]. Both *IGF1* and its receptor *IGF1R* were expressed in testicles, and their hormones act directly on male gonads [57,58]. In immature testes, *IGF1* promotes the development of sustentacular cells, Leydig cells and gonocytes. In mature testis, the *IGF1* gene induces spermatogenesis and regulates Leydig cell function [59,60]. *IGF1* may act as an autocrine/paracrine or endocrine signal to regulate testicular steroid production as well as germ cell and Sertoli cell functions [61]. *IGF1* plays different roles in testicular function at different stages of testicular development [54,62]. We speculate that high IGF1 and IGF1R protein amounts in the immature testis may suggest they highly promote the development and differentiation of sustentacular cells, Leydig cells and gonocytes in goat testis during sexual maturity.

Transcriptome data revealed the MAPK pathway is implicated in goat testicular development, while *TGFBR1*, *TGFB1*, *EGFR* and *IGF1* were enriched in this pathway and downregulated during sexual maturation, as key genes that regulate testis development and spermatogenesis [63,64]. Multiple reports suggest MAPK signaling is a critical regulator of testis growth and development, testis cell proliferation, differentiation and apoptosis and testosterone secretion, thus affecting male fertility [65,66,67]. One of the key downstream target genes of MAPK signaling is *IGF1*, which together with other genes in the pathway controls testis cell proliferation, testis volume development, hormone secretion and spermatogenesis, and is often reported to be associated with male fertility [55,68,69]. We speculate that MAPK signaling is a critical regulatory pathway in goat testis development and spermatogenesis. As an essential male fecundity-related gene in the MAPK signaling pathway, *IGF1* modulates goat testis growth and development and can affect the functions of various cells of goat testis by regulating other downstream genes in the signaling pathway. During male goat sexual maturation, *IGF1* regulates the development of the testis, spermatogenesis and hormone synthesis through the MAPK signaling pathway and other cooperative genes.

## 5. Conclusions

This study first used RNA-Seq to profile the expression of genes during testicular development in Qianbei Ma goats. We identified 544 genes with AS events between I and S, which suggests that AS of differential genes might be critical in the regulation of testicular development in goats. Totally, eight KEGG pathways were upregulated and 90 were downregulated between I and S. Totally, seven KEGG pathways were upregulated between S and P. Among the six screened DEGs (*TGFBR1*, *TGFB1*, *EGFR*, *IGF1*, *MAPK3* and *SMAD4*) *TGFBR1*, *TGFB1*, *EGFR*, *IGF1* and *MAPK3* belonged to the “MAPK signaling pathway”, corresponding to the GO term “protein phosphorylation”. However, the specific function and pathway mechanism of the selected genes still need to be further studied at the cellular level. The findings should further our understanding of gene regulation during testicular development and sperm formation. Our study also suggests that RNA-seq has great potential in the study of the transcriptome during the development of goat testis. 

## Figures and Tables

**Table 1:** Primers utilized in qRT-PCR.

Primer Name	Gene ID	Primer Sequence	Fragment Length	Annealing Temperature
GAPDH-F GAPDH-R	XM_005680968.3	ATGTTTGTGATGGGCGTGAA GGCGTGGACAGTGGTCATAAGT	153 bp	60 °C
TGFBR1-F TGFBR1-R	XM_018052233.1	TTCAAACGTGCTGACATCTATGC ACTGATGGATCGGAAGGTACAAG	128 bp	60 °C
SMAD4-F SMAD4-R	XM_018039535.1	CATAACAGCACTACCACCTGGACT GGATGATTAGAAATAGGAGGCTGG	173 bp	60 °C
TGFB1-F TGFB1-R	NM_001314142.1	CAACAATTCCTGGCGCTACCT ATGTCCACTTGAAGCGTGTTATCC	183 bp	60 °C
EGFR-F EGFR-R	XM_018067044.1	CCGTGCGATTCAGTAACAACC GGTCAATTTCTGGCAGTTCTCCTC	194 bp	60 °C
IGF1-F IGF1-R	NM_001285697.1	AATCAGCAGTCTTCCAACCCAA AGCAAGCACAGGGCCAGATA	114 bp	60 °C
MAPK3-F MAPK3-R	XM_018040780.1	CTGGACCGGATGTTGACCTTTA CTCCTTCAGTCGTTCCTTGGG	138 bp	60 °C

**Table 2:** Statistics of RNA-Seq data quality.

Sample Name	Library Number	Raw Reads (n)	Clean Reads (n)	Error Rate	Q20	Q30	GC pct
I1	1	40,405,582	39,128,916	0.03	97.36	93.09	50.99
I2	2	43,841,646	42,366,502	0.03	97.41	93.2	51.16
I3	3	39,433,208	38,237,518	0.03	97.42	93.25	51.42
I4	4	42,396,474	40,926,952	0.03	97.43	93.23	51.26
I5	5	45,463,988	43,854,898	0.03	97.41	93.19	51.1
I6	6	39,351,212	38,000,364	0.03	97.48	93.36	50.91
S1	7	44,934,642	43,739,660	0.03	97.51	93.41	52.09
S2	8	43,025,142	41,894,994	0.03	97.39	93.17	52.36
S3	9	45,845,898	44,647,422	0.03	97.59	93.55	52.15
S4	10	41,237,336	40,020,062	0.03	97.37	93.11	52.29
S5	11	41,130,946	39,901,252	0.03	97.36	93.09	51.09
S6	12	41,242,240	40,019,490	0.03	97.51	93.4	51.9
P1	13	45,377,178	44,131,516	0.03	97.48	93.34	52.27
P2	14	44,664,918	43,577,078	0.03	97.49	93.36	52.06
P3	15	43,792,502	42,601,198	0.03	97.47	93.32	51.98
P4	16	43,921,138	42,702,250	0.03	97.5	93.39	52.17
P5	17	43,223,452	42,134,186	0.03	97.44	93.27	52.14
P6	18	45,571,906	44,622,618	0.03	97.3	92.91	50.37

Error rate: overall sequencing error rate for the data; Q20 and Q30: percentages of total bases with Phred values above 20 and 30, respectively; GC pct: percentage of C and G among the 4 bases in clean reads.

**Table 3:** Statistics of reads aligned with the reference genome.

Sample	Total Reads	Total Map	Unique Map	Multi Map	Positive Map	Negative Map
I1	39,128,916	37,563,635 (96.00%)	35,626,251 (91.05%)	1,937,384 (4.95%)	17,793,049 (45.47%)	17,833,202 (45.58%)
I2	42,366,502	40,625,554 (95.89%)	38,612,044 (91.14%)	2,013,510 (4.75%)	19,294,015 (45.54%)	19,318,029 (45.60%)
I3	38,237,518	36,735,876 (96.07%)	34,685,761 (90.71%)	2,050,115 (5.36%)	17,323,488 (45.30%)	17,362,273 (45.41%)
I4	40,926,952	39,282,398 (95.98%)	37,389,786 (91.36%)	1,892,612 (4.62%)	18,672,350 (45.62%)	18,717,436 (45.73%)
I5	43,854,898	42,092,675 (95.98%)	40,255,938 (91.79%)	1,836,737 (4.19%)	20,105,549 (45.85%)	20,150,389 (45.95%)
I6	38,000,364	36,500,392 (96.05%)	34,501,227 (90.79%)	1,999,165 (5.26%)	17,230,278 (45.34%)	17,270,949 (45.45%)
S1	43,739,660	42,076,928 (96.20%)	40,551,689 (92.71%)	1,525,239 (3.49%)	20,259,699 (46.32%)	20,291,990 (46.39%)
S2	41,894,994	40,318,684 (96.24%)	39,006,285 (93.10%)	1,312,399 (3.13%)	19,484,346 (46.51%)	19,521,939 (46.60%)
S3	44,647,422	43,061,457 (96.45%)	41,514,183 (92.98%)	1,547,274 (3.47%)	20,739,259 (46.45%)	20,774,924 (46.53%)
S4	40,020,062	38,465,949 (96.12%)	36,829,538 (92.03%)	1,636,411 (4.09%)	18,396,451 (45.97%)	18,433,087 (46.06%)
S5	39,901,252	38,334,062 (96.07%)	36,942,595 (92.59%)	1,391,467 (3.49%)	18,452,256 (46.24%)	18,490,339 (46.34%)
S6	40,019,490	38,522,907 (96.26%)	36,912,252 (92.24%)	1,610,655 (4.02%)	18,439,977 (46.08%)	18,472,275 (46.16%)
P1	44,131,516	42,514,601 (96.34%)	41,073,585 (93.07%)	1,441,016 (3.27%)	20,518,750 (46.49%)	20,554,835 (46.58%)
P2	43,577,078	41,944,011 (96.25%)	40,303,516 (92.49%)	1,640,495 (3.76%)	20,133,389 (46.20%)	20,170,127 (46.29%)
P3	42,601,198	41,016,519 (96.28%)	39,440,892 (92.58%)	1,575,627 (3.70%)	19,701,050 (46.25%)	19,739,842 (46.34%)
P4	42,702,250	41,098,071 (96.24%)	39,528,322 (92.57%)	1,569,749 (3.68%)	19,745,068 (46.24%)	19,783,254 (46.33%)
P5	42,134,186	40,510,371 (96.15%)	38,849,502 (92.20%)	1,660,869 (3.94%)	19,405,731 (46.06%)	19,443,771 (46.15%)
P6	44,622,618	38,550,615 (86.39%)	37,036,723 (83.00%)	1,513,892 (3.39%)	18,501,103 (41.46%)	18,535,620 (41.54%)

Sample: sample name; total reads: number of clean reads upon quality control; total map: number (percentage) of reads aligned to the reference genome; unique map: number (percentage) of reads aligned to a unique region of ARS1.2 (subsequently analyzed for quantitation); multi map: number (percentage) of reads with alignment to many locations of ARS1.2; positive and negative maps: numbers (percentages) of reads with alignment to the positive and negative strands of the reference genome, respectively.

**Table 4:** Statistics of reads aligned to genomic regions.

Sample Name	Exonic Region	Intronic Region	Intergenic Region
I1	4,466,151,533 (79.48%)	611,822,545 (10.89%)	541,085,383 (9.63%)
I2	4,779,622,398 (78.65%)	761,383,464 (12.53%)	535,946,498 (8.82%)
I3	4,432,093,997 (80.66%)	570,346,947 (10.38%)	492,580,965 (8.96%)
I4	4,614,052,978 (78.53%)	738,532,433 (12.57%)	523,236,881 (8.90%)
I5	5,160,159,063 (81.96%)	605,739,527 (9.62%)	530,252,297 (8.42%)
I6	4,298,336,210 (78.73%)	599,791,632 (10.99%)	561,800,986 (10.29%)
S1	5,004,805,293 (79.49%)	606,545,683 (9.63%)	684,973,145 (10.88%)
S2	4,891,821,345 (81.09%)	533,206,687 (8.84%)	607,543,338 (10.07%)
S3	5,173,907,638 (80.30%)	600,212,860 (9.32%)	668,983,341 (10.38%)
S4	4,579,030,919 (79.56%)	552,189,052 (9.59%)	623,968,138 (10.84%)
S5	4,488,498,885 (78.27%)	613,561,446 (10.70%)	632,649,316 (11.03%)
S6	4,595,667,229 (79.73%)	543,550,434 (9.43%)	624,459,696 (10.83%)
P1	5,058,268,703 (79.52%)	636,322,977 (10.00%)	666,220,762 (10.47%)
P2	4,921,081,484 (78.42%)	644,761,157 (10.27%)	709,616,381 (11.31%)
P3	4,871,901,450 (79.39%)	593,069,117 (9.66%)	671,905,236 (10.95%)
P4	4,918,914,052 (80.00%)	601,562,419 (9.78%)	628,332,353 (10.22%)
P5	4,886,718,972 (80.63%)	554,456,620 (9.15%)	619,529,425 (10.22%)
P6	4,658,554,613 (80.75%)	537,138,992 (9.31%)	573,327,628 (9.94%)

Exonic, intronic and intergenic regions: numbers (percentages) of reads with alignment to the exonic, intronic and intergenic regions, respectively.

**Table 5:** Genes functions enriched through GO enrichment.

	S vs. I	P vs. S
BP	protein phosphorylation	sodium ion transport
	ion transport	metal ion transport
	transmembrane transport	monovalent inorganic cation transport
	reproduction	immune response
	reproductive process	immune system process
MF	ion channel activity	voltage-gated sodium channel activity
	channel activity	voltage-gated ion channel activity involved in regulation of postsynaptic membrane potential
	passive transmembrane transporter activity	sodium channel activity
	substrate-specific channel activity	chemokine activity
	scavenger receptor activity	chemokine receptor activity
CC	myosin complex	voltage-gated sodium channel complex
	extracellular matrix	sodium channel complex
	plasma membrane	ion channel complex
	plasma membrane part	cation channel complex
	cell periphery	integral component of plasma membrane

BP: biological processes; MF: molecular functions; CC: cellular components.

## Data Availability

The raw data from sequencing are available at NCBI under BioProject ID: PRJNA879963.
